# A Coupled Adsorption–Biodegradation (CAB) Process Employing a Polyhydroxybutyrate (PHB)–Biochar Mini Pilot-Scale Reactor for Trichloroethylene-Contaminated Groundwater Remediation

**DOI:** 10.3390/bioengineering12020148

**Published:** 2025-02-04

**Authors:** Laura Lorini, Marta Maria Rossi, Maria Letizia Di Franca, Marianna Villano, Bruna Matturro, Marco Petrangeli Papini

**Affiliations:** 1 Department of Chemistry, University of Rome, La Sapienza, Piazzale Aldo Moro 5, 00185 Rome, Italy; 2 Water Research Institute, IRSA-CNR, Via Salaria km 29,300, 00015 Monterotondo, Rome, Italy; 3 New Biodiversity Future Center, NBFC, Piazza Marina, 61, 90133 Palermo, Rome, Italy

**Keywords:** bioremediation, reductive dechlorination, adsorption, polyhydroxyalkanoates, biochar, dehalococcoides

## Abstract

Actions for improving water quality are critical and include the remediation of polluted groundwater. The effectiveness of the remediation strategy to remove contamination by chlorinated solvents may be increased by combining physicochemical treatments (i.e., adsorption) and biological degradation (i.e., biological reductive dechlorination (BRD)). Recent studies have shown the potentialities of bio-based materials for bioremediation purposes, including polyhydroxybutyrate (PHB), a biodegradable microbial polyester tested as a fermentable source of slow-release electron donors. Further, a low-cost biochar derived from the pyrolysis of pinewood waste (PWB), used as sorbent material, has recently been proposed to accelerate reductive microbial dehalogenation. Here, we propose a coupled adsorption and biodegradation (CAB) process for trichloroethylene (TCE) removal in a mini pilot-scale reactor composed of two reactive zones, the first one filled with PHB and the second one with PWB. This work aimed to evaluate the performance of the CAB process with particular regard to the effectiveness of the PWB in sustaining the biofilm, mostly enriched by *Dehalococcoides mccartyi*. The main results showed the CAB system treated around 1300 L of contaminated water, removing 102 mg TCE per day. Combining PHB and PWB had a positive effect on the growth of the dechlorinating community with a high abundance of *Dhc cells.*

## 1. Introduction

Chlorinated solvents such as perchloroethyelene (PCE) and trichlorethylene (TCE) are well-known soil and groundwater contaminants due to their widespread employment and improper storage. A practical way to remediate chlorinated aliphatic hydrocarbons’ (CAHs) matrices is based on the utilization of indigenous microorganisms able to “respire” these compounds [1]. These so-called organohalide-respiring bacteria (OHRB) can sequentially remove the chlorine atoms from the molecular carbon skeleton and replace them with a hydrogen atom [2]. This reaction, called biological reductive dechlorination (BRD), typically requires adequate redox conditions of the matrices and the presence of a considerable amount of molecular hydrogen, which represents the ultimate electron donor. The lack of these two conditions leads to the accumulation and storage of reductive dechlorination backbone daughter products, such as cis-dichloroethylene (cis-DCE) and vinyl chloride (VC), which can have a higher level of toxicity with respect to the original contaminant [3].

In order to prevent the occurrence of incomplete reductive dechlorination, engineered systems can be employed to provide electron donors or amendments directly in the subsoil. The enhanced in situ bioremediation (EISB) approach can be utilized to treat both dissolved plumes and sources, but a key factor is represented by the duration and utilization efficiency of the electron donor [4]. In this sense, the electron donor must be able to produce low H_2_ concentrations to avoid the occurrence of competitive reactions such as sulphate and nitrate reduction for a period long enough to allow OHRB growth [5]. For these reasons, in the last years, the utilization of slow-release electron donor sources, such as long-term fermentable materials [6], and the use of bioelectrochemical systems [7,8,9] are drawing interest from the scientific community. In this regard, our work aims to use, as a molecular hydrogen source, a biopolyester belonging to the class of polyhydroxyalkanoates (PHAs), which are biodegradable polymers naturally synthesized by microorganisms [10]. The polyhydroxybutyrate (PHB) utilized in our systems is produced by a biotechnological process through intracellular storage under unbalanced conditions of growth. In recent years, the use of PHB in groundwater remediation contexts has been evaluated, with good results in recent field studies [11]. Like all PHAs, PHB is completely biodegradable and, consequently, a source of organic acids and H_2_ [12]. On the other hand, biochar can be advantageously obtained from biomass residues with adsorption characteristics that depend on the applied thermal treatment [13]. Indeed, the RD reaction and the fermentation of a solid substrate for electron donor production have different rates, leading to low levels of efficiency due to the presence of different microbial competitive reactions (as extensively reported in the literature [14]). However, an interesting solution could be the immobilization of the solubilized contaminants on a highly physically supportive surface made from vegetable scrap biomass pyrolysis, which can provide different substantial improvements during EISB [15]. The first is the ability to distinguish the hydraulic retention time from the biomass retention time, assuring a sufficient contact time between the substrate and the microbial consortium that allows for the complete mineralization of the CAHs. In this view, the sorbent material supports dechlorinating biomass growth as biofilm, which is a more stable and change-resilient microbial configuration [16]. Another important effect is the instantaneous interruption of the contamination plume [17]. Hence, the CAB process allows for the continuous and sustained recovery of the sorption capacity, modifying the equilibrium due to the change in the adsorbed species [18,19]. In a previous study by our research group, a laboratory-scale experiment combined adsorption on biochar and the biodegradation of TCE sustained by lactate as an electron donor [19]. Starting from these promising results, we decided to equip a mini pilot-scale reactor, filled with a commercial PHB for long-term fermentation and biochar from pinewood (PWB) as sorbent material, in order to study the CAB process as a possible strategy for groundwater remediation. In fact, the configuration herein presented may simulate a permeable reactive barrier (PRB) section or represent a scalable reactor for ex situ treatment. The start-up phase of the mini pilot-scale experiment has been reported by Rossi et al. (2022), discussing the first 40 days of operation [20]. In the present study, the results from 8 working months of the CAB process are shown and discussed, together with the microbial characterization of the dechlorinating consortium.

## 2. Materials and Methods

### 2.1. Materials

#### 2.1.1. Biochar from Pine Wood (PWB)

The biochar was derived from pine wood wastes gasified at about 850 °C (Plößberg bei Tirschenreuth, Germany). Details of its production and morphological characteristics are reported in [21,22].

#### 2.1.2. Polyhydroxybutyrate (PHB)

A commercial PHB was used as an electron donor source. To allow for the long-lasting release of electron donors, two forms were mixed with soil (4% *w*/*w*): the powder form to ensure quick fermentation and the pellet form to guarantee a higher and consistent duration.

#### 2.1.3. Dechlorinating Enriched Inoculum Dehalococcoides (Dhc) 

The dechlorinating enriched culture was fed with TCE and lactate at a sludge retention time of 30 days, and its microbial composition has been previously reported [19]. Analytical methods used for monitoring chlorinated solvents’ biodegradation in the inoculum have been reported elsewhere [23,24].

#### 2.1.4. Anaerobic Mineral Medium

The mineral medium composition was as follows: 1 g/L NaCl, 0.5 g/L MgCl_2_·6H_2_O, 0.2 g/L KH_2_PO_4_, 0.3 g/L NH_4_Cl, 0.3 g/L KCl, 0.015 g/L CaCl_2_·2H_2_O, 0.05 g/L Na_2_S, 2.5 g/L NaHCO_3_, 1 mL/L metal solution [25], and 10 mL/L vitamin solution [26]:

### 2.2. Design of the PHB–PWB Reactor

The reactor consisted of a polymethylmethacrylate (PMMA) column. The central body was 144 cm long, and the inner diameter was 10 cm. The column was equipped on one side with 13 side gates wherein sampling ports with Mininert™ valves (VICI, Schenkon, Switzerland) were located. The reactor was designed to accommodate two different reactive zones, hereinafter called the “fermentative zone” and the “CAB zone”. The reactor configuration, the schematic setup, and the characteristics of the filling material are extensively reported in the previous study [20]. The schematic representation of the reactor is also reported in Figure S1, for simplicity.

#### 2.2.1. Feeding Solution and Working Conditions

The feeding solution was prepared by flushing the tap water with a gas mixture of N_2_/CO_2_ (70/30% *v*/*v*) to create the anoxic condition, then collecting the solution into a 25 L gas-tight self-collapsing Tedlar bag^®^ (Supelco, Cerritos, CA, USA) to avoid the headspace formation. TCE (ACS ≥ 99.5%, Sigma-Aldrich^®^, St. Louis, MO, USA) was spiked inside the bag through a septum (to achieve a final concentration of 0.1 mM, i.e., ≅14 mg/L). The feeding solution flowed from the bottom of the column with a VELP (Usmate Velate, MB, Italy) peristaltic pump. The effluent was also collected in a Tedlar bag^®^ to ensure that no release of toxic compounds into the environment occurred.

**Figure 1:**
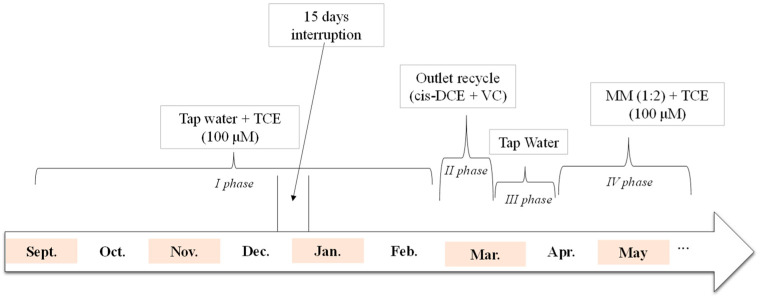
Schematic representation of the operative timeline.

Phase I corresponds to the start-up phase of the reactor and was conducted with a feeding solution consisting of tap water contaminated with TCE at a concentration of 0.1 mM. The overall duration was 165 days, during which 102 mg of TCE daily and a total of about 840 L of water were treated. At the end of this phase, the feeding solution was varied (Phase II) by recirculating the outlet solution from the top of the reactor (composed mainly of VC and, to a lesser extent, cis-DCE) without the addition of TCE. Under these conditions, about 60 L of water was treated in 15 days. Phase III was then performed by feeding the reactor only tap water. This period lasted 16 days and allowed for the treatment of about 70 L of water. In the last part of the experiment (Phase IV), the feeding consisted of mineral medium (MM) contaminated with 0.1 mM TCE. After 30 operative days, 68 mg of TCE per day and a total of about 300 L of water were treated. The theoretical operating conditions are reported in [20], and a schematic representation of the operative timeline is shown in Figure 1. However, the effective flow rate was calculated daily since the intrinsic fluctuations of the adopted pumping system must be considered. The results will therefore show the average values over the investigated period. Similarly, the actual incoming TCE (TCE in) concentration was determined by sampling when changing each feeding solution. The monitoring of CAHs and organic acids started after almost two hydraulic retention times (HRTs) of continuous feeding. On the other hand, the inlet and the outlet were sampled every day, whereas the profiles were performed once a week, both for the determination of CAHs and organic acids.

#### 2.2.2. Batch Tests

Once the column experiment was concluded, samples of the filling material (i.e., sand/PHB or sand/PWB) were taken from the two different zones of the reactor: PHA and biochar zones. Then, three series of batch tests were set up into 250 mL serum bottles in order to verify a possible limitation for the complete conversion of VC into ethylene, as described in Table 1.

A volume of 5 µL of TCE was then added to each test at the beginning and after the complete depletion of VC (around 90 days). CAHs and methane were determined twice a week by sampling from the headspace and directly analyzing the gaseous phase as reported in Section 2.3.

### 2.3. Analytical Methods

#### 2.3.1. Determination of CAHs in the Liquid Phase

To verify the correct input TCE concentration, the sample was taken through the septum of the Tedlar bag^®^ with a micro-glass syringe (Hamilton, Switzerland). On the other hand, the determination of the chlorinated intermediate was performed by taking 1 mL of the liquid phase from each side door with a plastic syringe and injecting it directly into a hermetically closed vial. The analysis was conducted on a (GC) Dani Master gas chromatograph (DANI Instruments, Contone, Switzerland) equipped with a flame ionization detector (FID), a capillary column (30 m × 0.53 mm ID × 3 um, TRB 624), and a DANI 86.50 headspace auto-sampler (Milan, Italy). The headspace analysis program was performed with an oven temperature of 80 °C, manifold at 120 °C, with the transfer line temperature at 180 °C. The vial was shaken softly for 1 min. Afterwards, the GC carrier gas (with flow of 10 mL/min) was He, the injector temperature was 180 °C (with split injection of 1:2), and the FID detector was at 200 °C with air, N_2_, and H_2_ (with flows of 240, 25, and 60 mL/min). To analyze TCE, cis-DCE, and VC, the oven temperature was programmed as follows: 60 °C for 3 min and 30 °C/min to 120 °C for 5 min.

#### 2.3.2. Determination of CAHs, Ethylene, and Methane in the Headspace

Chlorinated compounds (TCE and degradation products) and dechlorination by-products (e.g., ethylene and methane) were monitored during batch test experiments by sampling 50 μL of gaseous phase directly from the headspace of the serum bottles, using a glass gas tight syringe (Hamilton). Gaseous samples were directly injected into a GC Dani Master equipped with an FID detector and a Carbowax column (20 m × 2 mm) packed with Carbopack BDA 80/120 mesh. Helium (He) was used as carrier gas at a flow rate of 25 mL min^−1^, the injector temperature was set at 200 °C, and FID temperature was set at 200 °C. Temperature was programmed at 50 °C for 2 min and at 210 °C for 5 min, with a ramp rate of 20 °C per min.

#### 2.3.3. Determination of Organic Acids

The fermentation process was monitored by sampling about 2.5 mL of the liquid phase from the reactor for the analysis of volatile fatty acids (VFAs) (indeed, acetic acid and butyric acid are produced by the fermentation of PHB). For the analysis, the samples were filtered (0.22 μm Millex^®^ MCE syringe filter by Millipore) and analyzed using GC [12]. Briefly, 100 µL of oxalic acid (0.33 M) was added to 1 mL of the filtered sample, and 1 µL of the solution was injected into a GC Dani Master (Milano, Italy) equipped with a 2 m × 2 mm glass packed column in a Carbopack. The carrier gas flowed at 25 mL/min, the injector temperature was 200 °C, the oven temperature was 200 °C, and FID temperature was 200 °C.

#### 2.3.4. DNA Extraction and Quantification of Reductive Dechlorination Biomarkers by Digital Droplet PCR (ddPCR)

A total of 6 liquid samples (20 mL) for biomolecular analysis were collected from the PHB and biochar zones with the following operational phases: Phase I (tap water + TCE), Phase II (after outlet recirculation), and Phase IV (mineral medium + TCE). The samples were filtered using hydrophilic polycarbonate membranes (0.2 µm pore size, 25 mm diameter, Millipore) and immediately processed for genomic DNA extraction using the DNeasy PowerLyzer PowerSoil Kit (QIAGEN) according to the manufacturer’s instructions. A total of 6 samples (20 mL of slurry, consisting of mixed soil and mineral medium) from batch tests were collected and centrifuged at 20,000× *g* for 20 min. The supernatant was discarded, and total DNA extraction was performed on 1 g of the pellet obtained after centrifugation using the DNeasy PowerLyzer PowerSoil Kit (QIAGEN), following the manufacturer’s instructions.

The extracted DNA was used for the quantification of *Dehalococcoides mccartyi* (16S rRNA gene) and reductive dehalogenase genes (*tceA*, *bvcA*, and *vcrA*) via Digital Droplet PCR (ddPCR). Absolute quantification was performed using the QX200™ Droplet Digital™ PCR System (Bio-Rad, Hercules, CA, USA). For each sample, a PCR reaction mixture was prepared with a total volume of 22 μL, including ddPCR Supermix for Probes^®^ (Bio-Rad, USA), 3 μL of template DNA (dilution of 1:100), 900 nM of each primer, and 300 nM of the TaqMan probe [23]. Droplets were generated using the QX200 Droplet Generator (Bio-Rad, USA), and PCR amplification was performed on the T100 thermal cycler (Bio-Rad, USA). Quantitative data were subsequently obtained with the QX200 Droplet Reader (Bio-Rad, USA) and analyzed using QuantaLife software (Bio-Rad, USA). Data have been reported as gene copies per volume of liquid samples along with 95% confidence intervals.

## 3. Results and Discussion

### 3.1. TCE Removal via Reductive Dechlorination and Adsorption Processes

**Figure 2:**
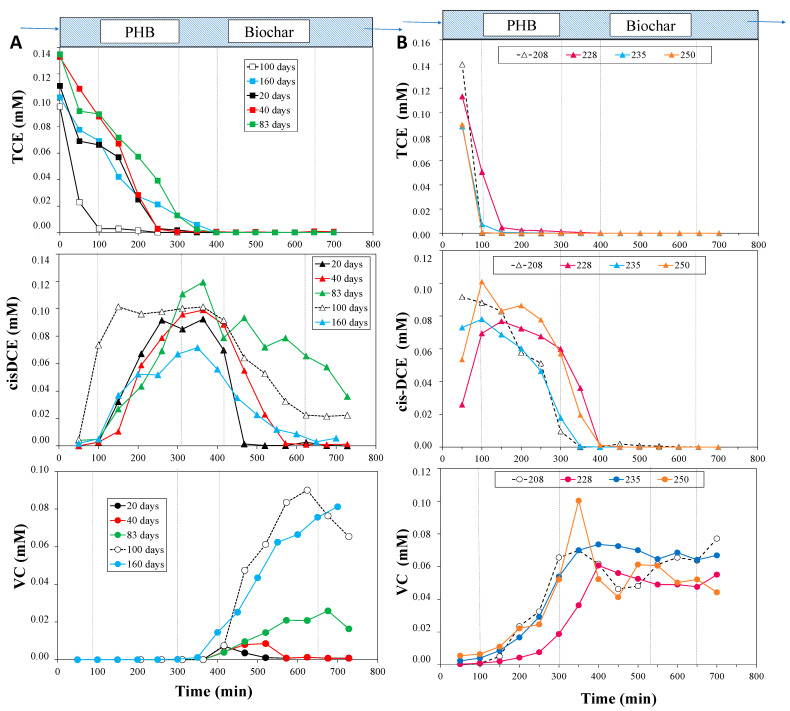
(**A**): TCE, cis-DCE, and VC concentration profiles along the column during the first 160 days of operation (Phase I); (**B**): TCE, cis-DCE, and VC concentration profiles along the column during days 208–250 (Phase IV).

**Figure 3:**
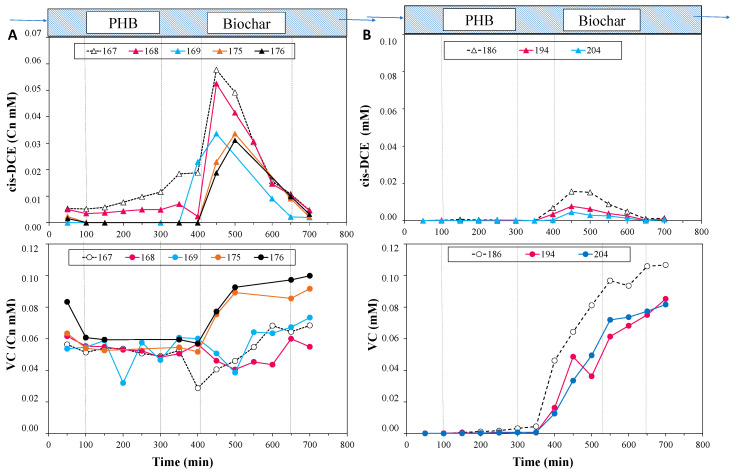
(**A**): cis-DCE and VC concentration profiles along the column during days 167–176 (Phase II); (**B**): cis-DCE and VC concentration profiles along the column during days 186–204 (Phase III).

**Figure 4:**
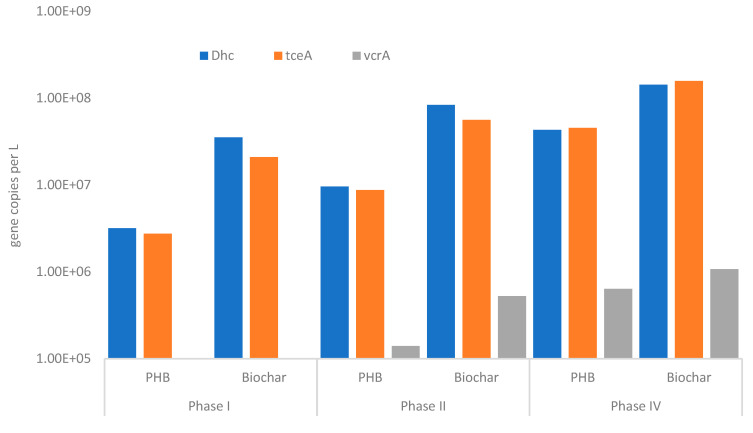
Abundances of *Dhc* and *tceA* genes estimated under different operating conditions: after feeding with tap water + TCE (Phase I), during recirculation from the outlet to the PHB zone of the reactor (Phase III), and after feeding with anaerobic mineral medium + TCE (Phase IV).

In the previous study [20], the start-up phase of the reactor is discussed. In detail, TCE and its daughter products’ (cis-DCE and VC) concentration profiles, as determined during the first 40 operative days, are illustrated. In order to better understand the TCE removal performances of the CAB system, the axial profiles of TCE and its daughter products have been reported during the first 160 days of the reactor’s operation (Phase I), from 167 to 176 days of operation (Phase II), from 186 to 204 days of operation (Phase III), and from 208 to 250 days of operation (Phase IV) (Figure 2 and Figure 3). The TCE profiles observed during Phase I with tap water and TCE feeding show that the concentration of TCE decreased in the fermentative zone filled with PHB. Then, in the biochar zone (after 300–400 min), the TCE was completely removed from the aqueous phase (Figure 2A). As pointed out in the previous study, during the first week of operation (data reported elsewhere [20]), the main contribution to TCE removal was made by the adsorption mechanism [22]. In contrast, after just 20 days, decreasing TCE profiles were observed along with the formation of cis-DCE and VC as byproducts of the biological reductive dechlorination, as also previously reported [20,27]. The quantification of reductive dechlorination biomarkers aligned with these findings (Figure 4). Specifically, a total of 3.53 × 10^7^ 16S rRNA *Dhc* gene copies/L was detected in the biochar zone, including 2.09 × 10^7^ copies/L of the *tceA* gene. Interestingly, *Dhc* (3.19 × 10^6^ 16S rRNA Dhc gene copies/L) was also detected in the fermentative zone, albeit at a lower level, despite the dechlorinating inoculum being introduced into the biochar zone. This finding suggests the occurrence of potential back-diffusion events, as previously reported by Rossi et al. (2022) [20].

In detail, Figure 2A shows the formation of cis-DCE, from 20 days of operation continuously until the last monitoring profile (160 days), in correspondence with the degradation of TCE in the very first sampling doors, with a peak in the maximum concentration at gate 7 (350 min). Hence, from gate number 8 (400 min), in correspondence with the beginning of the biochar zone, the cis-DCE concentration decreased. On the other hand, the formation of VC began after 20 days and significantly increased after 83 days of operation (Figure 2A). This behavior attests to the presence of dechlorinating culture activity. Moreover, until day 40, cis-DCE and VC were both depleted at gate 11 (after 550 min) thanks to the combined effect of BRD and adsorption on the biochar. However, after 83 days of operation, cis-DCE and VC were detected within the effluent, indicating that these intermediates had partially broken through the biochar. Indeed, from the cis-DCE profile at 83 days (Figure 2A), it is possible to highlight an increased concentration in the biochar zone (from gates 8 to 14), suggesting the reaching of the adsorption equilibrium of cis-DCE within the biochar. Then, after 100 days, the cis-DCE concentration significantly decreased. Therefore, the VC concentration increased with an opposite trend to that of the cis-DCE, reaching an average value of 0.08 ± 0.02 mM in the effluent. In this regard, it is noteworthy to underline the quantitative reduction of 0.1 mM of TCE fed to VC (Figure S2 shows TCE and its daughter products’ concentration in the effluent during the whole operative time, and after 100 days, the VC concentration settled at 0.08 ± 0.02 mM). As reported in Figure S2, during the last month of operation, VC was found in the effluent because of the combined effects of adsorption and biodegradation. However, ethylene, the final product of TCE reduction, was not detected either inside the reactor or in the effluent. As shown in Figure 4, only *Dhc* strains carrying *tceA* genes, which are known for the metabolic reduction of TCE to cis-DCE and VC and the co-metabolic reduction of VC to ethylene [28], were detected. In contrast, *bvcA* and *vcrA* genes were present at concentrations below 1 × 10^2^ gene copies/L (data not reported in the figure). These findings suggest that the residence time (1.8 days [20]) may have interfered with the dechlorination capabilities of the *Dhc* tceA-carrying strains, preventing the completion of the rate-limiting VC reductive dechlorination step. Additionally, it also may indicate a loss of selective pressure within the reactor for the *vcrA*- or *bvcA*-carrying strains capable of metabolic cis-DCE or VC dechlorination to ethylene, which were originally present in the inoculum [20]. This hypothesis was supported by microcosm studies conducted at the conclusion of the column reactor’s operation (see below).

Hence, the experiment was carried out by recirculating the effluent as the reactor feeding and starting operative Phase II, which lasted 15 days. In this way, the column was fed with a solution of VC (the main compound found in the column outlet) and a lesser concentration of cis-DCE. Figure 3A shows the concentration profiles along the column for cis-DCE and VC. The cis-DCE concentration increased in the biochar zone (from gate 8), indicating the conversion of the adsorbed TCE by the *Dhc* on the biochar zone. The VC concentration in the biochar zone and in the effluent increased too, as a consequence of the cis-DCE reduction. In line with these observations, after the recirculation of Phase II, the *Dhc* abundances increased both in the PHB zone (9.62 × 10^6^ 16S rRNA *Dhc* gene copies/L) and in the biochar zone (8.38 × 10^7^ 16S rRNA *Dhc* gene copies/L) and predominantly carried *tceA* genes (Figure 4). Interestingly, *vcrA* gene abundances also increased, reaching 1.4 × 10^5^ and 5.27 × 10^5^ *vcrA* gene copies/L in the PHB and biochar zones, respectively. Conversely, *bvcA* genes remained at concentrations below 1 × 10² gene copies/L.

At this point, Phase III started by feeding the reactor only tap water, in order to verify the possibility of regenerating the sorbent material by exploiting the dechlorinating microorganisms. Figure 3B shows that no chlorinated compounds were detected in the fermentative PHB zone, as expected, and contrary cis-DCE and VC concentrations increased from gate 8 (the biochar zone), suggesting that *Dhc*, which probably grew on the biochar like a biofilm, was converting the TCE molecules at the adsorption equilibrium on the biochar.

The last operative days (Phase IV) were then conducted by feeding the reactor with mineral medium contaminated with 0.1 mM TCE, in order to stimulate the biological response by supplying important nutrients (i.e., vitamins and metals) needed for biomass growth and metabolism. As shown in Figure 2B, the TCE concentration already decreased at gate 2 in the fermentative zone, with the consequent formation of cis-DCE and its complete depletion at the beginning of the biochar zone. Then, the VC concentration started increasing in the first part of the column, stabilizing at values ranging between 0.05 and 0.1 mM from gate 7 to 14 (in the effluent). These behaviors can be explained by the ability of *Dhc* biomass to rapidly respond to varying inputs (i.e., changes in feeding) and to the possibility that dechlorinating species may migrate to the fermentative zone, where electron donors are more readily available. As shown in Figure 4, at the end of Phase IV, the *Dhc* abundance increased by approximately one order of magnitude in both the PHB and biochar zones. This increase also included a corresponding rise in *tceA* and *vcrA* gene abundances. These findings further support the stimulating effect of the optimal conditions (e.g., the presence of additional nutrients in the medium) on the dechlorinating community within the column system, which had been progressively established over time under the various operational conditions tested.

During the entire working time, VFAs were monitored along the column to verify the fermentation of PHB as the slow release of electron donors (i.e., H_2_). Figure S3 shows that a constant VFA concentration of around 50 mg/L was detected along the entire reactor until day 201 of sampling. Subsequently, during Phase IV, the available VFA concentration increased to over 200 mg/L, probably as a consequence of the beginning of PHB pellet fermentation (in fact, the PHB powder form is more easily fermentable thanks to the higher specific surface area). Overall, it can be stated that PHB was able to sustain the BRD during the whole experiment, avoiding limitations due to the lack of electron donor [11,29].

### 3.2. TCE Reductive Dechlorination in Microcosms Derived from the Column System

The reductive dechlorination of TCE was further investigated in microcosms set up using material (PHB, Biochar) collected from the column system at the conclusion of the experimental phases. The microcosms were designed to replicate the conditions of the column system while extending the biomass retention time, thereby providing dechlorinating microorganisms with a longer time to complete the reductive dechlorination process. The experiments were conducted under various conditions including biostimulation with PHB or lactate, adsorption by biochar, and bioaugmentation with the same dechlorinating inoculum used for the start-up of the column system. These batch tests allowed us to better understand the possible kinetic or microbiological limitations that hindered the complete conversion of VC to ethylene.

**Figure 5:**
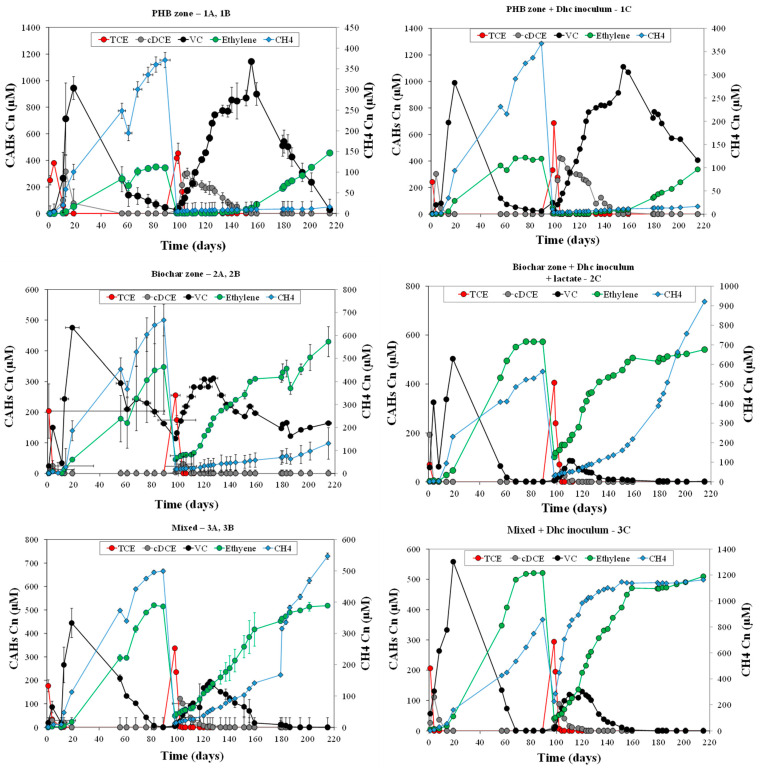
CAHs and ethylene concentration monitored during working days of batch tests, in comparison to methane concentration.

The complete reductive dechlorination of TCE to ethylene was observed under all tested conditions using materials collected from the following: (i) the fermentative zone filled with PHB (microcosms 1A and 1B) and further bioaugmented with the *Dhc* inoculum (microcosm 1C); (ii) the biochar zone (microcosms 2A and 2B) and further biostimulated with lactate and bioaugmented with the *Dhc* inoculum (microcosm 2C); and (iii) a mixture of both the PHB and biochar zones (microcosms 3A and 3B), further bioaugmented with the *Dhc* inoculum (microcosm 3C) (Figure 5).

Dechlorination in the microcosms containing biochar (microcosms 2 and 3) occurred more rapidly than in those without the sorbent material, which were set up using material from the fermentative zone (microcosm 1). In the latter, TCE was dechlorinated to VC, but the subsequent reduction of VC to ethylene was significantly slower compared to other microcosms, requiring approximately 80 days to complete. In contrast, in the microcosms containing material from the PHB zone (micrososms 2 and 3), the conversion of VC to ethylene occurred in approximately 60 days. This trend was further confirmed with the second TCE spike across all tested conditions.

**Figure 6:**
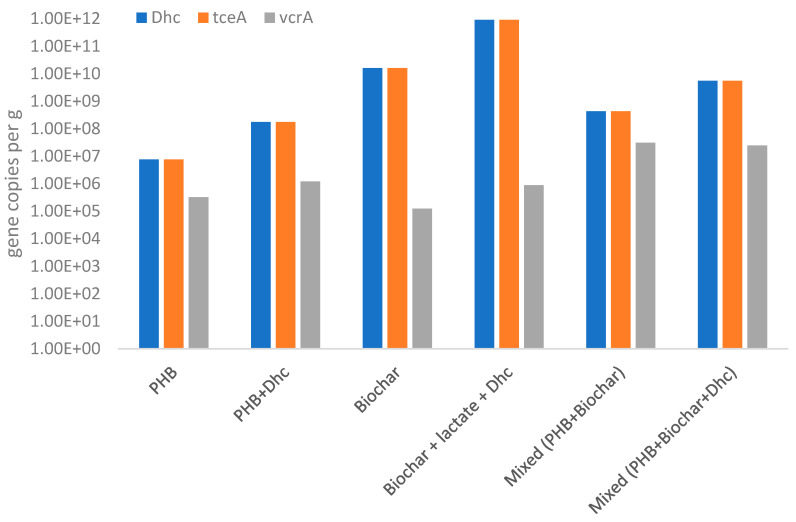
*D. mccartyi* (Dhc) and reductive dehalogenase gene (*tceA* and *vcrA* genes) abundances quantified at the conclusion of the batch tests.

The kinetic behaviors of the microcosms derived from the column system were supported by the observed abundance of reductive dechlorination biomarkers (Figure 6). *Dhc* was found to be highly abundant across all microcosm conditions, with *tceA* and *vcrA* identified as the predominant reductive dehalogenase genes. The biochar-based and mixed microcosms demonstrated the highest *Dhc* abundances, reaching up to 6.5 × 10¹⁰ *Dhc* 16S rRNA gene copies per gram of biochar, which further increased in the bioaugmented setups, as expected (Figure 6). Consistent with the observed complete TCE dechlorination, the *Dhc* abundances were notably high, likely reflecting the influence of retention time on the ability of *Dhc* cells to grow and complete the reduction of VC to ethylene. This process likely occurred through co-metabolic pathways in *Dhc tceA*-carrying strains or metabolic pathways in *Dhc vcrA*-carrying strains, which were already present in the column system but were previously unable to complete ethylene formation due to the limited retention time of the system. Additionally, batch tests suggested that biochar and bioaugmentation with *Dhc* inoculum significantly enhance the dechlorination process. This improvement is likely attributed to biochar’s capacity to act as a support for the formation of *Dhc* biofilms. Furthermore, methane production was observed across all conditions, highlighting the interplay between dechlorinating and fermentative microbial communities.

## 4. Conclusions

The study herein proposed combined two synergistic mechanisms which immobilize, biodegrade, and reduce the spread of contamination of TCE. In detail, the experiment conducted in a fixed-bed reactor allowed for a simulation of a condition close to a PRB or an external bioreactor for ex situ water treatment (with a reactive area consisting of biochar and dechlorinating biofilm). The mini pilot-scale reactor was continuously fed with an average 102 mg/day TCE and was able to remove over 99% of the fed TCE over the entire duration of the experiment (8 months), treating around 1300 L of water. To the best of our knowledge, this study is the first in the literature reporting a CAB process involving polyhydroxyalkanoates and biochar at the mini pilot scale for the dichlorination of TCE-contaminated water. The combination of PHB and PWB had a positive effect on the development of a specific dechlorinating community, characterized by a high abundance of *Dhc cells.* Although the conversion of VC to ethylene was not achieved throughout the entire operational period, the *Dhc* population established within the reactor was able to complete the dechlorination reactions, since *Dhc* strains carrying *tceA* or *vcrA* genes were already present in the system (as shown by microcosms) for a longer contact time. Moreover, the combination of PHB as an electron donor and PWB as a support for Dhc biofilm formation proved to be effective in facilitating the biological reductive dechlorination (BRD) processes. In this sense, the CAB process should allow for work to be performed under conditions that would normally exclude biological treatment (e.g., low residence times or a high pore water velocity). Future research should explore the role of biochar as a support for *Dhc* biofilm growth, the metabolic interactions among the microbial community members involved in fermentation and dechlorination pathways, and the potential to combine biochar derived from various sources (i.e., different waste streams) with polyhydroalkanoates produced from organic residues.

## Figures and Tables

**Table 1:** Description of three series of batch tests set by using the reactor filling material.

Series	A-B Tests (Replicates)	C Test (Single)
1	30 g of soil from the fermentative zone 150 mL of mineral medium	30 g of soil from the fermentative zone 150 mL of mineral medium 30 mL of dechlorinating biomass
2	30 g of soil from the reactive zone 150 mL of mineral medium lactate (5 mM)	30 g of soil from the reactive zone 150 mL of mineral medium lactate (5 mM) 30 mL of dechlorinating biomass
3	15 g of soil from the fermentative zone 15 g from the reactive zone 150 mL of mineral medium	15 g of soil from the fermentative zone 15 g from the reactive zone 150 mL of mineral medium 30 mL of dechlorinating biomass

## Data Availability

The original contributions presented in this study are included in the article/Supplementary Material. Further inquiries can be directed to the corresponding author(s).

## Supplementary Materials

The following supporting information can be downloaded at: https://www.mdpi.com/article/10.3390/bioengineering12020148/s1, Table S1: Pinewood Biochar characteristics; Figure S1: Scheme of the mini-pilot scale column reactor; Figure S2: TCE, cis-DCE and VC concentration in the effluent monitored during the operative time. The yellow dots represent TCE concentration in the feeding (TCE IN), reported here for comparison; Figure S3: Organic acids concentration determined along the column at different operative times.

## Abbreviations

BRD	Biological Reductive Dechlorination
CAB	Coupled Adsorption–Biodegradation
Cash	Chlorinated Aliphatic Hydrocarbons
cis-DCE	cis-Dichloroethelyne
Dhc	Dehalococcoide
EISB	Enhanced In Situ Bioremediation
FID	Flame Ionization Detector
GC	Gascromatograph
HRT	Hydraulic Retention Time
MM	Mineral Medium
OHRB	Organohalide-Respiring Bacteria
PCE	Perchloroethylene
PHA	Polyhydroxyalkanoates
PHB	Polyhydroxybutyrate
PMMA	Polymethylmethacrylate
PRB	Permeable Reactive Barrier
PWB	Pinewood Biochar
RD	Reductive Dechlorination
TCE	Trichloroethylene
TeCA	Tetrachloroethane
VC	Vinyl Chloride
VFA	Volatile Fatty Acid
