# Supplementary material for: A Coupled Adsorption–Biodegradation (CAB) Process Employing a Polyhydroxybutyrate (PHB)–Biochar Mini Pilot-Scale Reactor for Trichloroethylene-Contaminated Groundwater Remediation
Source: Bioengineering (Basel). 2025 Feb 4;12(2):148. doi: 10.3390/bioengineering12020148 (PMC11851903; doi:10.3390/bioengineering12020148)
Supplement: Supplementary file 1 [file bioengineering-12-00148-s001.zip › bioengineering-3417331-supplementary.pdf]

bioengineering

Supplementary Materials

A Coupled Adsorption–Biodegradation (CAB) Process Employing a Polyhydroxybutyrate (PHB)–Biochar Mini Pilot-Scale Reactor for Trichloroethylene-Contaminated Groundwater Remediation

Laura Lorini [1,*], Marta Maria Rossi [1], Maria Letizia Di Franca [2], Marianna Villano [1], Bruna Matturro [2,3] and Marco Petrangeli Papini [1]

1 Department of Chemistry, University of Rome, La Sapienza, Piazzale Aldo Moro 5, 00185 Rome, Italy; martamrossi@gmail.com (M.M.R.); marianna.villano@uniroma1.it (M.V.); marco.petrangelipapini@uniroma1.it (M.P.P.)
2 Water Research Institute, IRSA-CNR, via Salaria km 29,300, 00015 Monterotondo, Rome, Italy; marialetizia.difranca@irsa.cnr.it (M.L.D.F.); bruna.matturro@irsa.cnr.it (B.M.)
3 New Biodiversity Future Center, NBFC, Piazza Marina, 61, 90133 Palermo, Rome, Italy
* Correspondence: laura.lorini@uniroma1.it

Academic Editor: Dirk Holtmann

Received: 23 December 2024
Revised: 21 January 2025
Accepted: 1 February 2025
Published: 4 February 2025

Citation: Lorini, L.; Rossi, M.M.; Di Franca, M.L.; Villano, M.; Matturro, B.; Petrangeli Papini, M. A Coupled Adsorption–Biodegradation (CAB) Process Employing a Polyhydroxybutyrate (PHB)–Biochar Mini Pilot-Scale Reactor for Trichloroethylene-Contaminated Groundwater Remediation. *Bioengineering* **2025**, *12*, 148. https://doi.org/10.3390/bioengineering12020148

Copyright: © 2025 by the authors. Licensee MDPI, Basel, Switzerland. This article is an open access article distributed under the terms and conditions of the Creative Commons Attribution (CC BY) license (https://creativecommons.org/licenses/by/4.0/).

Table S1. Pinewood Biochar characteristics [1]

Specific Surface Area (m^2 g^{-1})	Total Pores Volume (cm^3 g^{-1})	Micro-pores Volume (cm^3 g^{-1})	q_{max} (mg g-1)
343 ± 2	0.383	0.136	109.41 ± 5.62

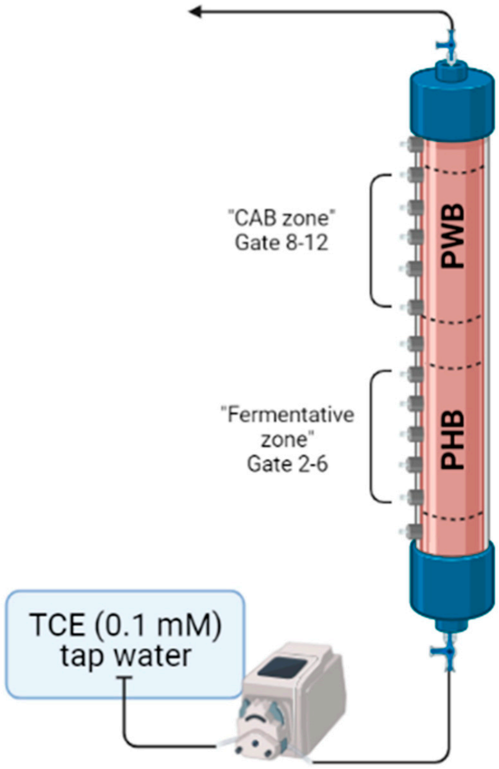

Figure S1. Scheme of the mini-pilot scale column reactor [2].

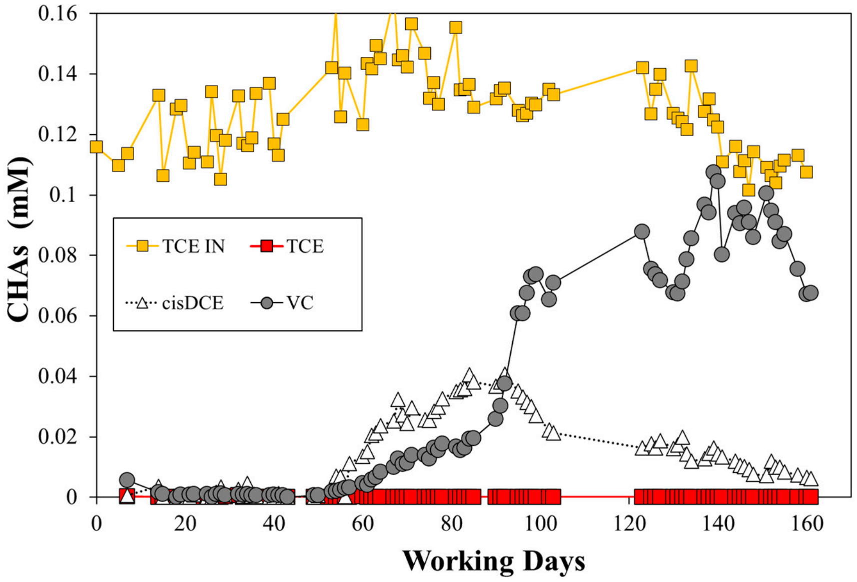

Figure S2. TCE, cis-DCE and VC concentration in the effluent monitored during the operative time. The yellow dots represent TCE concentration in the feeding (TCE IN), reported here for comparison.

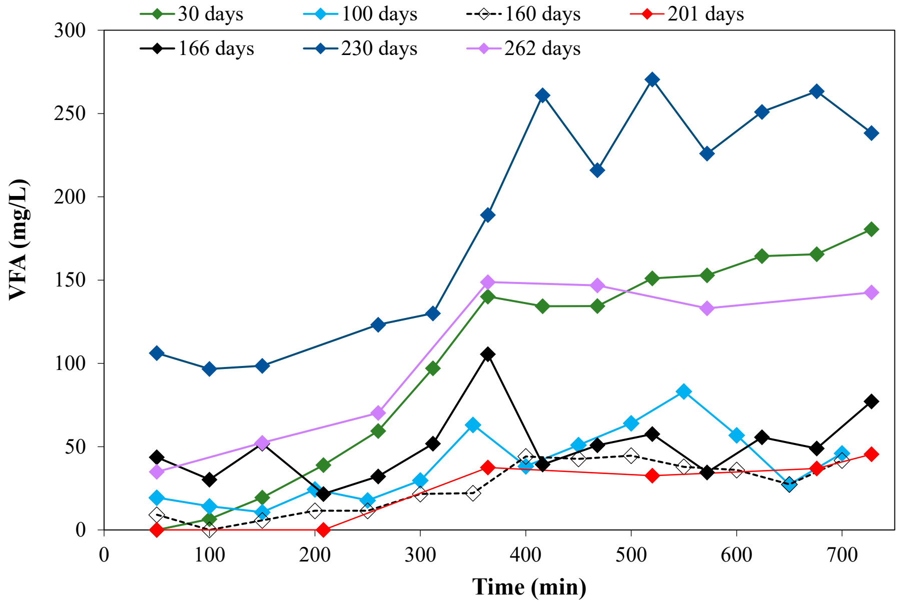

Figure S3. Organic acids concentration determined along the column at different operative times.

References

1. Rossi, M.M.; Silvani, L.; Amanat, N.; Petrangeli Papini, M. Biochar from Pine Wood, Rice Husks and Iron-Eupatorium Shrubs for Remediation Applications: Surface Characterization and Experimental Tests for Trichloroethylene Removal. *Materials* **2021**, *14*, 1776, doi:10.3390/ma14071776.
2. Rossi, M.M.; Alfano, S.; Amanat, N.; Andreini, F.; Lorini, L.; Martinelli, A.; Papini, M.P. A Polyhydroxybutyrate (PHB) -Biochar Reactor for the Adsorption and Biodegradation of Trichloroethylene : Design and Startup Phase. *Bioengineering* **2022**, *9*.

Disclaimer/Publisher's Note: The statements, opinions and data contained in all publications are solely those of the individual author(s) and contributor(s) and not of MDPI and/or the editor(s). MDPI and/or the editor(s) disclaim responsibility for any injury to people or property resulting from any ideas, methods, instructions or products referred to in the content.
